# Long-term fitness effects of the early-life environment in a wild bird population

**DOI:** 10.1093/beheco/araf097

**Published:** 2025-09-26

**Authors:** Yuheng Sun, Terry A Burke, Hannah L Dugdale, Julia Schroeder

**Affiliations:** Groningen Institute for Evolutionary Life Sciences, University of Groningen, Nijenborgh 7, Groningen 9747 AG, the Netherlands; School of Natural Sciences, Faculty of Science and Engineering, Macquarie University, Sydney, New South Wales 2109, Australia; Ecology and Evolutionary Biology, School of Biosciences, University of Sheffield, Sheffield, S10 2TN, United Kingdom; Groningen Institute for Evolutionary Life Sciences, University of Groningen, Nijenborgh 7, Groningen 9747 AG, the Netherlands; Department of Life Sciences, Imperial College London, Silwood Park, Ascot, Berkshire, SL5 7PY, United Kingdom

**Keywords:** aging, competition, noise, predictive adaptive response, senescence, silver spoon

## Abstract

Environmental conditions and experiences during development can have long-term fitness consequences, including a reduction of adulthood survival and reproduction. These long-term fitness consequences may play an important role in shaping the evolution of life history. We tested two hypotheses on the long-term fitness effects of the developmental environment—the silver spoon hypothesis and the internal predictive adaptive response (PAR) hypothesis. We compared the change in annual survival and annual reproductive output with age for adult birds hatched and/or reared in poor––impacted by anthropogenic noise, and/or high sibling competition––and good––not impacted by anthropogenic noise, and/or low sibling competition––environments. We used a 23-year longitudinal dataset from a wild house sparrow (*Passer domesticus*) population inhabiting an isolated island, which enabled near-complete monitoring and unusually accurate lifetime fitness estimates. We used a cross-fostering setup to disentangle environmental effects experienced postnatally from those experienced prenatally. We found that adults that, as chicks experienced more within-brood competition had a stronger increase in early-life annual survival, but also a stronger decrease in late-life annual survival. Females that hatched in a noisy environment produced fewer genetic recruits annually, supporting a sex-specific silver spoon hypothesis. Males reared in a noisy environment had accelerated reproductive schedules, supporting a sex-specific internal PAR hypothesis. Our results highlight that anthropogenic noise (∼68 dB from power generators) can have long-term fitness consequences in wild animals, altering their life-history strategies, and that effects may be sex-specific.

## Teaser

Growing up in a noisy environment can affect animals for life. However, the details of these effects are still debated. We found that wild female house sparrows experiencing noise during early life produced fewer offspring and males who grew up in noisy environments aged faster. Even birds exposed to noise while they were still in the egg were affected. Our results highlight the lasting impacts of human-made noise on wildlife.

## Introduction

Early-life environments can have profound impacts on individual fitness (Lindström 1999), including rates of senescence (the decline in survival and reproduction in later life; Kirkwood and Austad 2000; Cooper and Kruuk 2018). The silver spoon hypothesis proposes that organisms developing in an unfavorable environment bear lifetime fitness costs because the environmental circumstances constrain the development of an optimum phenotype (Grafen 1988; Monaghan 2008). The predictive adaptive response (PAR) hypothesis proposes that organisms can respond to environmental cues at the developmental stage and thus maximize their lifetime fitness for the given circumstances (Gluckman and Hanson 2004; Gluckman et al. 2005; Nettle et al. 2013). Both the silver spoon and the PAR hypotheses recognize the detriment of early-life adversity, but they make different predictions about how individuals respond to early-life adversity. Here, we test both the silver spoon and the internal PAR hypotheses by examining how early-life environmental adversity influences age-specific survival and reproduction across the lifespan.

The silver spoon hypothesis predicts that individuals that experience a better early-life environment have higher fitness than those from a worse early-life environment (Grafen 1988). Support for the silver spoon hypothesis is widely found across taxa, including humans (Hales and Barker 2001; Wu et al. 2010) and other animals (Lindström 1999). However, fitness components can respond to early-life environmental conditions in a sex-specific (Sanghvi 2021) or age-specific (Spagopoulou et al. 2020; Crosland et al. 2022) manner. A meta-analysis found no evidence that the early-life environment affected survival senescence in the wild, but there was a small silver spoon effect of the early-life environment on reproductive senescence (Cooper and Kruuk 2018).

The internal PAR hypothesis provides an alternative perspective on how early-life environment shapes later-life survival and reproduction. The internal PAR (also known as the Future Lifespan Expectation) hypothesis proposes that early-life adversity results in a soma that has reduced survival probability at any age; thus it is advantageous for the individual to adjust their reproductive schedule according to their reduced expected lifespan, to maximize their fitness (Wells 2012; Nettle et al. 2013). Therefore, the internal PAR predicts that individuals from adverse early-life environments will exhibit accelerated reproductive schedules. Empirical studies testing the internal PAR are less abundant than for the silver spoon hypothesis and have found mixed results, with some supporting it (Berghänel et al. 2016) but others not (Weibel et al. 2020; van de Crommenacker et al. 2022).

Testing the silver spoon and internal PAR hypotheses on the long-term effects of the early-life environment, especially under wild conditions is important for understanding the evolution of life history and senescence. This is because in captivity, environmental conditions are usually controlled, therefore the fitness measures do not reflect the real fitness consequences in the wild (Monaghan 2008) and these fitness consequences can play important roles in life-history evolution and population dynamics (Lindström 1999). However, testing these hypotheses in the wild is difficult due to the significant challenge in obtaining complete life-history data with known age and longevity for sufficient individuals that survive to old age. Those few studies that have overcome these difficulties have provided important insights into early-life environmental effects in wild populations (Drummond et al. 2011; Ancona and Drummond 2013; Cartwright et al. 2014). However, as they rely on observational data and natural variation, we miss information on the causality of such effects. In contrast, by experimentally fostering chicks between nests, our study can directly test for whether the early-life environment causally affects life-history traits, or whether individuals of different quality breed in early-life environments of matching quality. Cross-fostering allows environmental effects to be disentangled from genetic effects and offers a more rigorous framework for understanding the role of early-life environmental conditions in shaping fitness-related traits.

The impacts of anthropogenic noise on wildlife are well documented, ranging from alterations in individual behavior to shifts in ecological communities (Shannon et al. 2016). Importantly, exposure to anthropogenic noise has been linked to accelerated telomere attrition (Meillère et al. 2015; Injaian et al. 2019), reduced body condition (Ware et al. 2015), and impaired somatic development (de Soto et al. 2013; Nedelec et al. 2015), suggesting the potential for long-term fitness consequences. This is supported by recent longitudinal work showing that early-life noise exposure can reduce lifetime reproductive output in birds (Meillère et al. 2024). In house sparrows, noise may disrupt parent-offspring acoustic communication, thereby reduce parental feeding rates and contributing to poor nestling condition (Schroeder et al. 2012b). However, it remains unclear whether early-life noise exposure leads to lasting effects on survival and reproduction, or whether it induces changes in reproductive scheduling across the lifespan.

Our study tests the silver spoon and the internal PAR hypotheses in a natural study system that has complete life-history data, and allows postnatal environmental effects to be disentangled from genetic effects through cross-fostering, in a closed, long-term-monitored population of house sparrows (*Passer domesticus*) on Lundy Island in the United Kingdom. Immigration and emigration are extremely rare (Schroeder et al. 2015), which allowed us to track every individual until their death. House sparrows are cavity-nesting, peridomestic small passerine birds, and we can closely monitor their breeding events in nest boxes. Therefore, the Lundy house sparrow system provides us with access to complete life-history data, including lifetime fitness (Alif et al. 2022). Secondly, cross-fostering experiments have been routinely conducted in this population for 23 years. By cross-fostering chicks right after hatching, we can separate the effects of the postnatal environment from the effects of prenatal factors, including genetic factors. Thirdly, a proportion of breeding sites provide a poor early-life environment as the parents have reduced provisioning rates due to the impact of chronic background noise (Schroeder et al. 2012b). Chicks reared in this poor environment have lower fledgling success, lower body mass at fledging, and lower recruiting success than chicks reared in a quiet environment (Schroeder et al. 2012b), demonstrating that the noisy environment is an adverse early-life environment and leads to a poor somatic state. Additionally, clutch size differs among broods regardless of the environment. Although parents are expected to optimize the brood size to their resource providing ability, variation in the number of chicks that survive to fledge may introduce variation in the intensity of within-brood competition (Schroeder et al. 2012b), and large brood sizes are associated with reduced chick mass (Cleasby et al. 2011). Both, the noise presence and the social competition can induce early-life adversity in birds (Nur 1984; Injaian et al. 2019), allowing us to test the silver spoon and internal PAR hypotheses in this wild population.

**Fig. 1.**
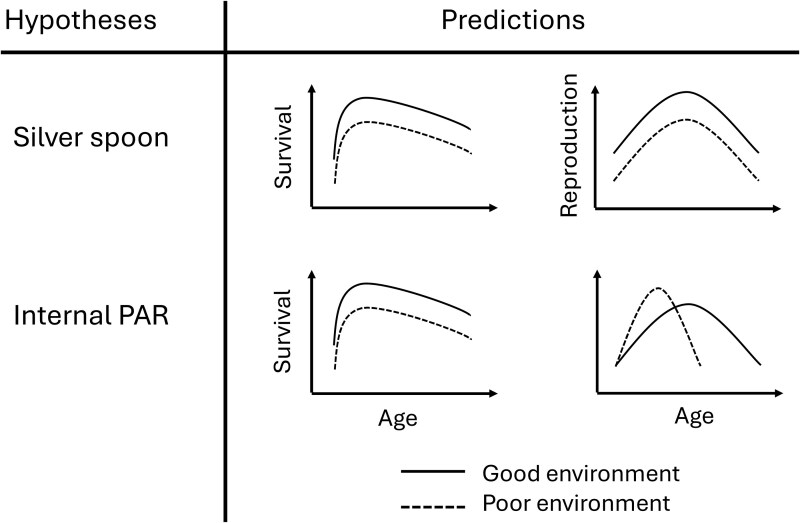
Predictions of the silver spoon and the internal predictive adaptive response (PAR) hypotheses in relation to survival and reproductive success.

In this study, we investigate survival and reproduction across ages in response to the presence of environmental noise and the number of early-life competitors in Lundy house sparrows, to test the silver spoon and internal PAR hypotheses. (1) The silver spoon hypothesis predicts that an adverse developmental environment has overall negative effects on fitness components; thus, sparrows reared in a noisy environment and/or with more intense within-brood competition are expected to have lower annual adulthood survival and lower annual reproductive output compared with sparrows reared in a quiet environment and/or with less intense within-brood competition. (2) The internal PAR hypothesis predicts that birds reared in an adverse developmental environment have lower annual adulthood survival, earlier reproductive peaks and higher initial rates of increase and then steeper declines in reproductive success (Fig. 1). This study will therefore contribute to our understanding of the effects of early-life environment on long-term fitness in wild populations.

## Methods

### Study population

The Lundy house sparrows are free-living and resident on Lundy Island (51 °10′N, 4 °40′W), United Kingdom. Lundy is 19 km from the nearest land, and because house sparrows are not well suited for long-distance flights (Bengtson et al. 2004), the immigration and emigration rates are low (ca. 0.5% immigration rate and three confirmed emigrants 2000 to 2015; Schroeder et al. 2015). A female can produce 0 to 6 broods per breeding season (2.3 ± 0.9, Westneat et al. 2014). They usually lay one egg per day, with the number of eggs ranging from 1 to 7 (4.2 ± 0.8, Westneat et al. 2014). Eggs are hatched on the same day (Cleasby et al. 2010). The mean hatching success is 0.70, resulting in the number of hatchlings ranging from 0 to 7 (2.8 ± 1.6). Coefficient of variance for chick mass per brood measured on day 2 or 3 is 0.23 ± 0.12. The mean fledging success is 0.36, resulting in the number of fledglings ranging from 0 to 5 (1.0 ± 1.3).

Since 2000, the Lundy house sparrow population has been systematically monitored. Nest boxes and known natural nests have been checked routinely during the breeding season for reproductive activities. Once a nest is complete, it is checked every second day for eggs. Therefore, precise laying dates are known. Nests with eggs are left with minimal disturbance until the 12th day after laying, after which they are checked every day to record precise hatching dates and brood sizes (Cleasby et al. 2010). Nests are revisited on the 12th day after hatching, and chicks that survive to this age are considered fledglings (Cleasby et al. 2010). Sightings and captures have been carried out throughout the breeding season (April–August) and for 1 or 2 weeks in the winter. Each bird was ringed with a unique combination of three color rings and a numbered metal ring from the British Trust for Ornithology. More than 99% of birds were ringed as nestlings, fledglings or in their first year, and are therefore of known age (Schroeder et al. 2015). The annual probability of re-sighting an individual was 0.96 (95% CI 0.95 to 0.97, Simons et al. 2019).

### Early-life environmental noise

**Fig. 2.**
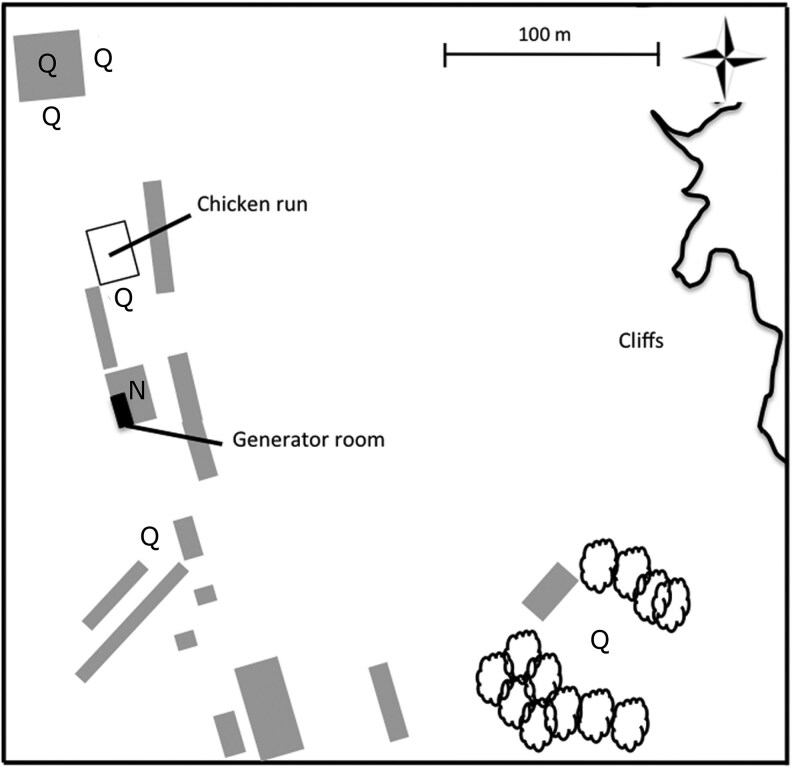
Locations of the house sparrow nest boxes on Lundy island. Q and N represent quiet and noisy areas, respectively. The chicken run is the main feeding site of the house sparrows. Modified from Schroeder et al. (2012b).

Lundy Island is not connected to the national power grid. Since March 2001, a set of generators has been running 06:00 to 24:00 h daily (Schroeder et al. 2012b). These generators produce low-frequency noise that reverberates in the surrounding area—a workshop that is semi-enclosed with stone walls and corrugated roofing, with a permanently open gate and a louvered window that allows the birds access. This physical structure reverberates noise within the workshop but restricts the noise reverberation outside the workshop. The noise level within the workshop was significantly higher than it was outside the workshop (Schroeder et al. 2012b). In our analyses, “noisy environment” refers to nests located inside the workshop, while “quiet environment” refers to nests located elsewhere on the island. Figure 2 shows the locations of quiet and noisy areas in relation to the generators and the main feeding site. Nestbox occupancy, parental body mass, age and reproductive investment did not significantly differ between noisy and quiet areas, suggesting no association between parent quality and the noisy area (Schroeder et al. 2012b). Both the workshop and the surrounding village and farm buildings contain nest boxes that have fluctuated in number over the past 23 years. In 2008, there were 29 house sparrow nest boxes in the workshop (noisy) and 101 nest boxes elsewhere (quiet) (Schroeder et al. 2012b), and in 2023 the numbers were 28 and 88, respectively. In response to finding that house sparrow parents provision their offspring less often in noisy conditions (Schroeder et al. 2012b), noise reduction measures were implemented in the workshop in 2013, lowering average noise levels from 68 dB(A) to 45 dB(A) (Schroeder et al. 2012b and measurement by YS in 2023, respectively). Therefore, the dataset used in this study only contained birds hatched before 2013 to keep the early-life environmental noise constant. The dataset was then restricted to birds that had been seen after fledging to focus on the long-term effects of early-life environmental noise. This excludes the noise-related reduction in offspring survival between hatching and fledging (Schroeder et al. 2012b).

### Cross-fostering

Chicks were routinely cross-fostered whenever there were enough same-aged broods, with the exceptions of 2008 and 2010, when the breeding population was so small that broods of the same age were not available (Winney et al. 2015). Most chicks were cross-fostered at 2 days old, except that in 2000 to 2003, cross-fostering took place at 3 days old (Winney et al. 2015). While this may have led to the exclusion of years with particularly poor breeding conditions, such exclusion may also have prevented these extreme contexts from masking the effects of noise and within-brood competition in the developmental environments. Broods were cross-fostered entirely or partially, but the original brood size was always left unchanged after cross-fostering. Chicks remained in their foster broods until fledging. Chicks were cross-fostered either within or between noisy and quiet areas opportunistically, depending on where the same-age broods were located. Only cross-fostered individuals were included in this study. The final dataset included 1,057 individuals. Sample sizes for individuals cross-fostered between quiet and noisy environments (eg, quiet-to-quiet, quiet-to-noisy) are provided in Table S7. The environments where the bird hatched and where they were fostered were hereafter referred to as the natal and rearing environment, respectively.

### Annual survival

Annual survival data were generated as follows: a bird was observed in a given year if it met any one of these criteria: (1) it was sighted, (2) it was captured, or (3) it had a reproductive record (eg genetic pedigree indicated that it produced offspring in that year). Out of the 1,057 individuals, 31 were last observed by re-sighting, 910 were last observed by a capture, and 116 were last observed through social or genetic parentage. When a bird was not observed for two consecutive years, it was considered to have died in the year following the last observation. For example, if a bird was observed in 2005, but not in 2006 and 2007, it was considered to be alive in 2005 and dead in 2006. Following this process, all birds in the dataset were classified as dead by the time this dataset was assembled (December 2023), and thus had complete lifespans and lifetime data. The annual survival dataset contained 1,687 observations from 1,057 birds. Lifespan in this dataset ranged from 0 to 9 y (1.6 ± 1.2). Less than 0.8% of the birds in this population reappear after not being observed for 2 years.

### Annual reproductive output

We used the number of annual genetic recruits as a measure of annual reproductive fitness for all birds that survived until the breeding season following their year of hatching. The number of genetic recruits is the best proxy of long-term fitness (Alif et al. 2022), so we used this measure to quantify the long-term fitness consequences caused by the early-life environment. A genetic recruit of a focal bird is defined as an individual that (1) is the genetic offspring of the focal bird and (2) has been assigned its own offspring in the genetic pedigree. The pedigree was constructed using Cervus 3.0 with up to 23 microsatellite markers (Marshall et al. 1998; Dawson et al. 2012), and contained 9,057 individuals hatched in 1995 to 2019. The number of annual recruits was first matched to the annual survival data. If a bird survived but had no recruits in a year, zero annual recruits were assigned to the bird for that year. The annual reproductive output dataset contained 274 observations from 133 females, and 356 observations from 165 males.

### Statistical analyses

All statistical analyses were conducted in R 4.3.2 (R Core Team 2023).

#### Adult survival

To test the silver spoon and the internal PAR hypotheses on survival, the effects of early-life environmental noise and social competition on survival were tested using a Generalized Linear Mixed Model (GLMM) with a binomial distribution, using *glmmTMB* 1.1.8 (Brooks et al. 2017). An initial model was built with annual survival as the response variable (0 = dead, 1 = survived), and the fixed effect of presence or absence of noise in the rearing environment (0 = noisy, 1 = quiet), presence or absence of noise in the natal environment (0 = noisy, 1 = quiet), the number of fledglings from the brood where the focal bird was reared (continuous), the number of hatchlings from the brood where the focal bird was reared (continuous), sex (0 = female, 1 = male), natal brood order (the chronological order of breeding attempts within the same breeding season, continuous), and foster brood order (continuous). We also included age in years as we were interested in changes in ageing over time, and age^2^ was included to allow nonlinear changes with age. The presence or absence of noise in the natal environment was included to control for prenatal environmental noise effects. The number of hatchlings in the rearing brood was included to control for the quantity of resources that parents provided to a brood, because the parents' provisioning frequencies are positively associated with the number of chicks being fed (Schroeder et al. 2012b). For chicks born in broods with the same numbers of hatchlings, higher fledging numbers may reflect stronger or more prolonged competition among nestmates, although parental provisioning may adjust following early brood reduction (Ploger 1997). We included the brood orders of natal and foster broods (1st to 5th) to control for potential effects of breeding time and physiological costs of previous breeding attempts, which may influence offspring condition and survival (Verhulst et al. 1997; Verhulst and Nilsson 2008). Age, number of fledglings, and number of hatchlings were centered prior to modeling.

The initial model included all interactions of age and age^2^ with the natal environment, rearing environment, and the number of fledglings to test whether the early-life environment effect of these factors changed the shape of the senescence trajectory. The interactions of sex with age and age^2^ were included to control for sex differences in senescence, which are common in vertebrates (Clutton-Brock and Isvaran 2007). Additionally, an interaction between natal and rearing environments was included to test whether a bird hatched in a noisy environment would be less affected by the noise in the rearing environment than a bird hatched in a quiet environment. Males and females can respond to environmental factors differently due to morphological or behavioral dimorphism (Marasco et al. 2019; Sanghvi 2021; Mainwaring et al. 2023), therefore the interactions of sex with natal environment, rearing environment, and number of fledglings were included.

We included bird ID as a random effect to account for the nonindependence of the annual survival data across years for the same individual. Year was included as a random effect because mortality can be year-dependent due to environmental variations such as temperature (MJ Simons et al. preprint from bioRxiv.org). Natal brood ID was included because individuals hatched in the same brood might share genetic factors that influence fitness (Schroeder et al. 2012a); foster brood ID was included because individuals reared by the same pair of parents might share parental-care-related factors that also influence fitness (Ivimey-Cook et al. 2023).

To confirm any age effect detected by the survival model actually described a decrease during a sparrows' lifetime, a post-peak analysis was run using a GLMM, with the dataset restricted to data points after the peak using the following method: annual survival peaked at centered age = 1.7 years, thus observations with centered age > 1.7 years were used to build a new GLMM (*n* = 162); the structure of the new GLMM was the same as the final survival model, except that terms involving age^2^ were removed to inspect only the linear relationship between annual survival and age after the peak.

#### Reproductive success

To test the silver spoon and the internal PAR hypotheses on reproductive success, we used two sex-specific GLMMs. Females and males were modeled separately because the reproductive biology and reproductive senescence differ between the sexes (Schroeder et al. 2012a). In both sexes, the model was fitted with a negative binomial distribution, ensuring the ratio of observed and predicted zeros is within the tolerance range (checked by *performance* 0.13.0, Lüdecke et al. 2021). The initial models were built with the number of annual genetic recruits as the response variable. We included the same early-life effects as in the survival model: noise in the rearing and foster environments, the number of fledglings and hatchlings in the rearing brood and the number. We included an indicator for the last reproductive event (0 = no, 1 = yes) to control for terminal effects (ie the last reproduction of birds may have increased or decreased output, Bouwhuis et al. 2009; Schroeder et al. 2012a). We included mean age as mean of the ages that each individual appeared in the dataset, and Δage = age − mean age. With mean age and Δage in the same model, mean age captured the between-individual effect (eg differences in average reproductive output among individuals that tend to live longer), and Δage captured the within-individual effect (eg effects of senescence or experience, van de Pol and Wright 2009). This decomposition allows us to separately test for selective disappearance and within-individual changes across age. Δage^2^ was included to allow nonlinear changes with Δage. We again added the brood orders of the foster and rearing broods. The initial models included all interactions of each of Δage and Δage^2^ with natal environment, rearing environment, and number of fledglings, as well as the interaction between natal and rearing environments. The reproduction models included the same random effects structure as the survival model. In the female reproduction model, the random effect natal brood ID caused model convergence problems, and was removed as it was associated with problematic eigenvalues indicated by the *diagnose* function in *glmmTMB*.

After the initial models were run, nonsignificant interactions were removed, removing the least significant first until only significant interactions remained. This aids interpretation of first-order effects. Estimates and relevant statistics of removed terms at each step can be found in Table S8-10. The fit of the final models was evaluated by visually inspecting the residual plots generated by *DHARMa* 0.4.6 and ensuring all residual tests performed by the *simulateResiduals* function were passed (Hartig 2024). Collinearity was checked, ensuring that the VIF for all fixed effects was <3 (Zuur et al. 2009). Likelihood ratio tests were performed for the final models, and all significant effects detected by the models were confirmed. To ensure the conclusion was not affected by pseudoreplication due to the removal of natal brood ID in the female reproduction model, a data subset containing only one randomly sampled bird from each natal brood was tested again using the same model.

## Results

### Adult survival

**Fig. 3.**
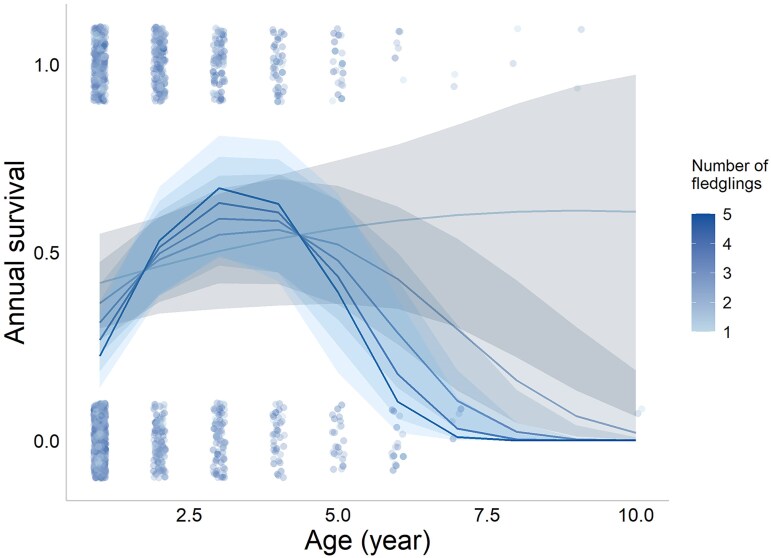
Annual adult survival in relationship to age and the number of fledglings in Lundy house sparrows. Each dot represents an observation, jittered to aid visualization; lines are predicted annual survival rates for different numbers of fledglings; shaded areas represent 95% confidence intervals; color saturation indicates the intensity of within-brood competition.

In the final adult survival model, the interaction of the number of fledglings in the foster brood and age^2^ was statistically significant (Table 1). Adult annual survival first increased then decreased with age except for individuals reared in broods with only one fledgling where there was no later decrease (Fig. 3). The more competitors a bird had as a nestling, the steeper were its initial increase and later decrease in adult survival (Fig. 3). Neither in the natal nor the rearing environment did noise show significant effects (Table 1). Adult annual survival in males was higher than in females (Table 1). For results of the full model, see Table S1. The post-peak analysis did not detect a decline in adult annual survival in late life (Table S2).

**Table 1.** Estimates from the final GLMM explaining annual adult survival by age in Lundy house sparrows.

Fixed effects	Level	Estimate	Std. error	*z*	*P*
(Intercept)	…	−0.100	0.318	−0.315	0.753
Age	…	0.632	0.095	−0.888	<0.001
Age^2^	…	−0.184	0.031	1.468	<0.001
Natal environment	Quiet	0.117	0.157	0.746	0.456
Rearing environment	Quiet	0.035	0.156	0.222	0.824
Number of hatchlings	…	0.019	0.069	0.268	0.789
Number of fledglings	…	0.010	0.079	−3.820	0.896
**Sex**	**Male**	**0.270**	**0.119**	**2.266**	**0.023**
Natal brood order	…	−0.096	0.102	−0.940	0.347
Foster brood order	…	−0.097	0.102	−0.952	0.341
Age × Number of fledglings	…	0.249	0.080	3.736	0.002
**Age^2^** **×** **Number of fledglings**	**…**	**−0.094**	**0.024**	**−3.915**	**<0.001**
**Random effects**	**1,687 observations**	**Variance**	**…**	**…**	
Bird ID	1,057 individuals	<0.001	…	…	
Year	22 years	0.561	…	…	
Natal brood ID	459 natal broods	0.180	…	…	
Foster brood ID	449 foster broods	<0.001	…	…	

Levels and corresponding sample sizes for categorical effects: natal environment: noisy = 351, quiet = 1,336; rearing environment: noisy = 359, quiet = 1,328; sex: female = 797, male = 890. Significant highest-order fixed effects are in bold.

### Reproductive success

**Fig. 4.**
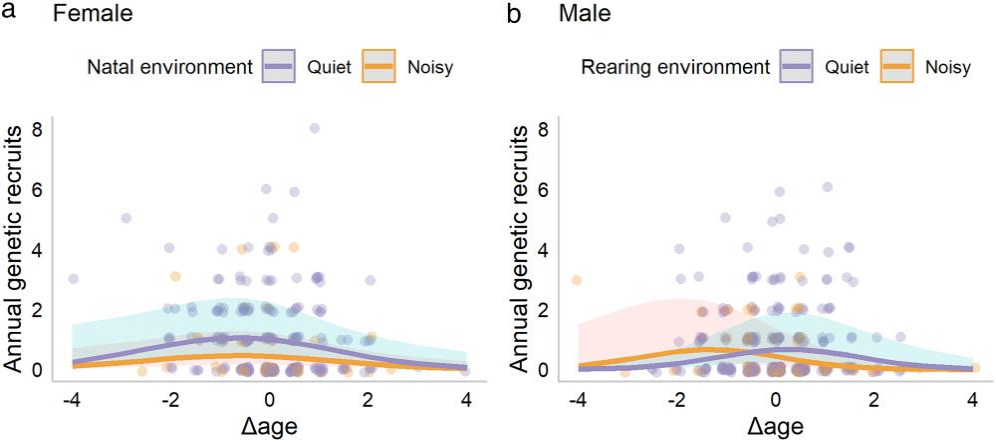
The number of annual genetic recruits in relationship to within-individual-centered age of adult Lundy house sparrows. The mean ages of birds hatched in a noisy (orange) and quiet (purple) environment were: 1.8 years and 2.0 years for females, and 2.2 years and 2.0 years for males, respectively. Each dot represents an observation; lines are predicted number of annual genetic recruits; shaded areas represent 95% confidence intervals. (a) Females hatched in a noisy environment produced fewer genetic recruits than females hatched in a quiet environment. (b) Males reared in a noisy environment exhibited earlier reproductive peaks relative to their lifespan than those reared in a quiet environment (0.9-year-old versus 2.3-year-old, respectively).

In both, females and males that survived to at least 1 year of age, the number of annual genetic recruits first increased and then decreased with within-individual-centered age (Fig. 4). Adult females hatched in a noisy environment produced fewer annual genetic recruits than adult females hatched in a quiet environment, regardless of their age; but the noise in the rearing environment did not show a significant effect (Table 2, Fig. 4a). The significantly positive effect of mean age implies selective a disappearance of low-quality adult females (Table 2). The interaction between rearing environment and Δage was removed because it was not significant. The significant effects of natal environment and mean age were confirmed in the subset where only one bird from each natal brood was modelled to account for pseudoreplication (Table S4).

**Table 2.** Estimates from the final GLMM explaining the number of annual genetic recruits by age in adult Lundy house sparrows.

Fixed effects	Level	Estimate	Std. error	*z*	*P*	Estimate	Std. error	*z*	*P*
	…	Female				Male			
(Intercept)	…	−3.693	0.803	−4.596	<0.001	−2.412	0.756	−3.189	0.001
Number of hatchlings	…	0.218	0.117	1.859	0.063	0.034	0.111	0.305	0.761
Number of fledglings	…	−0.068	0.109	−0.625	0.532	0.080	0.109	0.733	0.464
Natal environment	Quiet	**0.821**	**0.341**	**2.409**	**0.016**	−0.114	0.271	−0.423	0.672
Rearing environment	Quiet	0.458	0.252	1.819	0.069	0.431	0.322	1.339	0.181
Δage	…	−0.169	0.102	−1.661	0.097	−0.649	0.261	−2.482	0.013
Δage^2^	…	**−0.130**	**0.056**	**−2.307**	**0.021**	**−0.246**	**0.077**	**−3.209**	**0.001**
Natal brood order	…	0.018	0.176	0.105	0.917	−0.029	0.186	−0.155	0.877
Foster brood order	…	0.090	0.168	0.537	0.591	−0.141	0.170	−0.833	0.405
Last reproduction	Yes	0.401	0.223	1.800	0.072	0.352	0.248	1.423	0.155
Mean age	…	**0.709**	**0.142**	**5.007**	**<0.001**	**0.749**	**0.161**	**4.658**	**<0.001**
Rearing environment × Δage	Quiet	**…**	**…**	**…**		**0.773**	**0.274**	**2.815**	**0.005**
**Random effects**	**…**	**274 observations**	**…**	**…**	**Variance**	**356 observations**	**…**	**…**	**Variance**
Bird ID	…	133 individuals	…	…	<0.001	165 individuals	…	…	0.151
Year	…	20 years	…	…	0.335	21 years	…	…	0.518
Natal brood ID	…	…	…	…		148 natal broods	…	…	<0.001
Foster brood ID	…	111 foster broods	…	…	0.098	148 foster broods	…	…	<0.001

Levels and corresponding sample sizes for categorical effects: natal environment: noisy = 53 (female) and 58 (male), quiet = 221 (female), and 298 (male); rearing environment: noisy = 69 (female) and 58 (male), quiet = 205 (female) and 298 (male); last reproduction: no = 159 (female) and 222 (male), yes = 115 (female) and 134 (male). Significant highest-order fixed effects are in bold. Δage: within-individual-centered age.

Adult males reared in a noisy environment had an earlier peak in their number of annual genetic recruits produced, relative to their lifespan (Table 2, Fig. 4b). The peak for adult males reared in a noisy environment was 1.3 years before the within-individual mean age and for adult males reared in a quiet environment was 0.3 years after. The difference in the timing of the peak was confirmed by bootstrapping (mean difference = 1.6 years, *P* < 0.001). Given that the mean ages of the noisy and quiet groups were 2.2 years and 2.0 years, respectively, the peaks translated into 0.9 years in the noisy group and 2.3 years in the quiet group in chronological age, confirming that adult males reared in a noisy environment had an earlier peak in their annual reproductive output than those reared in a quiet environment. Noise in the natal environment did not have a significant effect (Table 2). The significantly positive effect of mean age implied selective disappearance of low-quality adult males (Table 2). The number of fledglings from the rearing brood did not have a significant effect on the reproduction of females and males that survived the first year of life (Table 2). For results of the full models, see Table S3.

## Discussion

We found sex-specific support for the silver spoon hypothesis in females, and for the internal PAR hypothesis in males, for annual reproductive success but not for survival, in a wild population using a multiyear cross-foster experiment.

### Adult survival

We found no support for the silver spoon or the internal PAR hypothesis in our annual adult survival analysis. Since only individuals that survived the first year of their life were included in our analysis, the negative effects of a poor early-life environment might be masked by selective disappearance during the first year of life, given that chicks reared in a noisy environment were less likely to fledge and recruit (Schroeder et al. 2012b), confirming meta-analytic results (Cooper and Kruuk 2018). The absence of the effect on survival could also result from adaptive physiological mechanisms that primarily conserve survival-enhancing traits during development at the cost of reproduction-enhancing traits (Cooper and Kruuk 2018), but further studies are needed to investigate this hypothesis.

We found an interactive effect of the number of fledglings on the rates of change in annual adult survival: while statistically controlling for the number of hatchlings, for each additional fledgling in the brood, the quadratic effect of age increased by 0.094 units per year (Table 1). This means that when there was one fledgling from the brood, the change of survival chance with age was mild, but as the number of fledglings increased, the age-related variation became larger (Fig. 3). In this population, a chick's final mass was negatively associated with brood size, suggesting poorer body condition at fledging for birds reared in larger broods (Cleasby et al. 2011). Fledglings in poor body condition could have lower chances of surviving the post-fledging period compared with those in good condition (Perrig et al. 2017). It is possible that in our study, the birds that underwent more intense within-brood competition had worse survival right after fledging, but they could improve their body condition to a level of average or above average in mid-life, at the cost of getting fragile more rapidly in late life. To confirm this effect on the decline rate, we ran a post-peak analysis which, however, did not detect a significant decline in late-life survival (Table S2). This is possibly due to the small sample size of individuals that survived to age four (162 observations from 84 individuals), and the sample sizes for very small or large numbers of fledglings were even smaller (13 observations from 6 individuals for number of fledglings = 1, 10 observations from 4 individuals for number of fledglings = 5). Another possible explanation for the interactive effect of the number of fledglings is that the larger broods might have a larger variation in fledgling quality, and low-quality individuals were quickly eliminated from the population, leading to the reduction of survival right after fledging, while high-quality individuals performed better than average throughout their life. Future studies may examine post-fledging survival in relation to fledgling numbers and consider within-brood variation when investigating its causes.

### Reproductive success

We found sex-specific support for the silver spoon hypothesis and the internal PAR hypothesis with annual reproductive success. The silver spoon effect on annual reproductive success was only observed in females, but it was the noise in their natal environment that reduced their reproductive success, whereas no effect of noise in their rearing environment was detected (Fig. 4a). In contrast, the internal PAR was only observed in males, where the noise in their rearing environment accelerated their reproductive schedule (Fig. 4b).

Noise in the natal environment had an overall negative effect on female annual reproductive output but not in males. Wild-derived zebra finches (*Taeniopygia guttata castanotis*) also experience a negative effect on reproductive output of prenatal environmental noise, but in both sexes (Meillère et al. 2024). The deleterious effects of prenatal environmental noise also included reductions in embryonic survival and telomere lengths (Meillère et al. 2024). An experiment in domestic chickens (*Gallus gallus domesticus*) also showed that prenatal chronic noise exposure was associated with a decrease in body and brain development (Kesar 2014). All this implies that noise affects embryos in eggs. Alternatively, it could also be the exposure in the first 2 days after hatching that played a role, given that we cross-fostered the chicks at the age of 2 days. In wild blue-footed boobies (*Sula nebouxii*), females that experienced El Niño southern oscillation during prenatal and natal stages showed early recruitment and altered relationship between age and laying date, respectively, whereas second-year experience affected male breeding success (Ancona and Drummond 2013). Taken together with our findings, this pattern suggests sex-specific developmental sensitivity in birds: female reproduction appears more susceptible to environmental conditions during embryonic and immediate postnatal stages, whereas male reproductive performance is more influenced by environmental cues encountered later in development. This sex-specificity could be explained by prenatal environmental disturbances interfering with female germ cell development, given that ovary and female germ cell development occur during incubation, while male germ cell development only takes place after sexual maturation (Aire 2014; Johnson 2014).

Poor egg quality caused by malnutrition in mothers breeding in the noisy area was unlikely an explanation in our study system because the noisy area was relatively close to the main feeding site compared to other breeding areas, and mothers' body mass did not differ between noisy and quiet areas (Fig. 2, Schroeder et al. 2012b). However, the noise could still lead to reduced egg quality through maternal pathways, for example, by disturbing their sleep behavior (Grunst et al. 2023), incubation behavior (Viigipuu et al. 2023), and/or increasing oxidative stress and altering hormones (Injaian et al. 2018; Flores et al. 2019). Future studies examining egg composition such as hormone levels, antioxidants, or yolk nutrients will help clarify whether the prenatal effects observed in this study are mediated through changes in egg quality. Our dataset only included individuals that survived the first year of life. Thus, our results do not detect selective disappearance occurring in early life, ie our dataset is biased to only those individuals that did survive to the second year, which are potentially of higher quality. Despite this, we found a negative effect of prenatal environmental noise on female reproductive output, suggesting this effect to be strong and still acting later in life. However, we could not separate this prenatal environmental effect into genetic and environmental components. Although the overall parental body mass, age and reproductive investment did not differ between quiet and noisy areas, older males (0.3 years difference) were more likely to breed in the noisy area, which, again assuming selective disappearance, means that fathers in the noisy area could be of better quality (Schroeder et al. 2012b). However, such a genetic effect would only reduce or neutralize the negative effect of the noise; thus, the negative effect of the prenatal environment detected here is a conservative estimate.

Males reared in a noisy environment exhibited a reproductive peak more than a year earlier than those reared in a quiet environment (Fig. 4b). This is rare empirical support for the internal PAR hypothesis compared with previous studies, which did not find effects (eg Weibel et al. 2020; van de Crommenacker et al. 2022) or where the effects were explained by other aspects such as growth rates, motor skill acquisition and immune function (Berghänel et al. 2016), instead of accelerated reproduction (but see Ancona and Drummond 2013 and Cartwright et al. 2014). However, it remains unknown why we only observed this response in males, but not in females. One possibility is that the response in females was masked by selective disappearance in the first year, and the same selection was not as strong in males. Future studies may investigate sex-specific selective disappearance in relation to rearing environments.

Theoretically, postponed reproduction will only evolve when the fitness gain from the increase in annual reproductive success with age outweighs the fitness loss due to annual mortality. Given the high annual mortality (∼0.5, Fig. 3) and the mild increase in annual reproductive output with age (Fig. 4) in Lundy house sparrows, the fitness benefit of postponing reproduction is in theory unlikely. However, variation in the age of reproductive peak was observed in this and a previous study (Schroeder et al. 2012a), suggesting a potential benefit of postponing reproduction. This could be because mortality varies largely by year due to environmental effects, and thus the plasticity in the timing of reproduction could be beneficial as it allows the birds to survive harsh years and breed in favorable years (MJ Simons et al. preprint from bioRxiv.org).

### Anthropogenic noise

In wild animals, anthropogenic noise is associated with reduced immediate reproductive success (Halfwerk et al. 2011; Kight et al. 2012), greater short-term telomere attrition (Meillère et al. 2015; Injaian et al. 2019), altered hormone levels (Injaian et al. 2018; Flores et al. 2019), and reduced body condition (Ware et al. 2015). However, studies focusing on the long-term effects of the noise are rare, except for one study in crickets *Teleogryllus oceanicus* (Gurule-Small and Tinghitella 2019) and one in zebra finches (Meillère et al. 2024 ) that found negative effects. Our study shows that the impact of anthropogenic noise experienced in early life can last to late life and lead to negative lifetime fitness consequences and alteration in life history strategy. This long-term impact on wildlife should be considered when discussing noise-pollution-related questions in urbanization.

## Conclusion

Using data from a wild bird population, our study found support for the sex-specific silver spoon effect: a reduction in the annual reproductive output of females hatched in an adverse environment. We also found support for sex-specific internal PAR: accelerated reproductive schedules in males reared in an adverse environment. We demonstrate that anthropogenic noise up to and including the first 2 days of life can affect female birds' long-term fitness, and shift reproductive schedules in males. We highlight that noise pollution can have long-term impacts on wild animals' fitness and alter their life history strategies.

## Supplementary Material

araf097_Supplementary_Data

## Data Availability

Analyses reported in this article can be reproduced using the data provided by Sun et al. (2025).
