# Correction to: Long-term fitness effects of the early-life environment in a wild bird population

**DOI:** 10.1093/beheco/araf145

**Published:** 2026-01-22

This is a **correction** to:

Yuheng Sun, Terry A Burke, Hannah L Dugdale, Julia Schroeder, Long-term fitness effects of the early-life environment in a wild bird population, *Behavioral Ecology*, Volume 36, Issue 5, September/October 2025, araf097, https://doi.org/10.1093/beheco/araf097

In the originally published version of this manuscript, figure 1 is missing, and subsequently the numbering of the remaining figures and their in-text references is incorrect.

This error has been corrected.

