# Supplementary material for: Long-term fitness effects of the early-life environment in a wild bird population
Source: Behav Ecol. 2025 Sep 26;36(5):araf097. doi: 10.1093/beheco/araf097 (PMC12477671; doi:10.1093/beheco/araf097)
Supplement: araf097_Supplementary_Data [file araf097_supplementary_data.zip › BE_Sun_supplemental_final.docx]

**Long-term fitness effects of the early-life environment in a wild bird population**

**Supplemental methods:** unusual individuals

The senescence curves were potentially skewed by two extreme individuals, displayed in Figure 2 (the two oldest individuals) and Figure 3 (the only female and male that had data at Δage = -4, -3, 3 and 4). They are a female and a male that both lived for nine years, which is a rare long lifespan for wild house sparrows. Therefore, the analyses were rerun excluding these two individuals from the dataset. For the annual adult survival model, the interaction between age^2^ and number of fledglings was no longer significant. Instead, the interaction between age and number of fledglings and the interaction between age^2^ and natal environment became significant (Table S2).

For the reproduction models, the negative effect of noise in the natal environment on female annual genetic recruits remained significant (Table S3, Figure S1), but the effect of noise in the rearing environment on male annual genetic recruits disappeared (Table S3). Because we are confident that they are true data points, we kept these two individuals in the dataset and interpreted the models accordingly, but we highlight that the negative effect of the noise in the natal environment on female reproduction is the most robust effect and not driven by extreme long-lived individuals.

**Table S1.** Estimates from the full GLMM (before removing insignificant interactions) explaining annual adult survival by age in Lundy house sparrows. Levels and corresponding sample sizes for categorical effects: natal environment: noisy = 351, quiet = 1,336; rearing environment: noisy = 359, quiet = 1,328; sex: female = 797, male = 890. Significant highest-order fixed effects are in bold.

| **Fixed effects** | **Level** | **Estimate** | **Std. Error** | **z** | **P** |
| --- | --- | --- | --- | --- | --- |
| (Intercept) |  | 0.181 | 0.406 | -0.445 | 0.656 |
| Age |  | 0.441 | 0.263 | 1.678 | 0.093 |
| Age^2^ |  | 0.062 | 0.096 | -0.650 | 0.516 |
| Natal environment | Quiet | -0.253 | 0.353 | 0.719 | 0.472 |
| Rearing environment | Quiet | -0.103 | 0.353 | 0.292 | 0.770 |
| Number of hatchlings |  | 0.031 | 0.070 | 0.440 | 0.660 |
| Number of fledglings |  | -0.032 | 0.109 | -0.290 | 0.772 |
| Sex | Male | -0.172 | 0.350 | -0.491 | 0.623 |
| Natal brood order |  | -0.102 | 0.103 | -0.984 | 0.325 |
| Foster brood order |  | -0.077 | 0102 | -0.756 | 0.449 |
| Age × Natal environment | Quiet | 0.311 | 0.220 | 1.409 | 0.159 |
| Age × Rearing environment | Quiet | 0.042 | 0.195 | 0.214 | 0.831 |
| Age × Number of fledglings |  | 0.213 | 0.089 | 2.405 | 0.016 |
| Age × Sex | Male | -0.102 | 0.150 | -0.682 | 0.495 |
| Age^2^ × Natal environment | Quiet | -0.082 | 0.082 | -0.991 | 0.322 |
| Age^2^ × Rearing environment | Quiet | -0.102 | 0.072 | -1.411 | 0.158 |
| Age^2^ × Number of fledglings |  | -0.065 | 0.035 | -1.875 | 0.061 |
| Age^2^ × Sex | Male | -0.006 | 0.360 | -0.411 | 0.909 |
| Natal environment × Rearing environment | Quiet × Quiet | -0.155 | 0.360 | -0.432 | 0.666 |
| Natal environment × Sex | Quiet × Male | 0.210 | 0.301 | 0.698 | 0.485 |
| Rearing environment × Sex | Quiet × Male | 0.360 | 0.302 | 1.194 | 0.232 |
| Number of fledglings × Sex | Male | 0.004 | 0.128 | 0.031 | 0.975 |
| **Random effects** | **1,687 observations** | **Variance** |  |  |  |
| Bird ID | 1,057 individuals | <0.001 |  |  |  |
| Year | 22 years | 0.549 |  |  |  |
| Natal brood ID | 459 natal broods | 0.181 |  |  |  |
| Foster brood ID | 449 foster broods | <0.001 |  |  |  |

**Table S2** Estimates from the GLMM post-peak analysis for annual adult survival by age in Lundy house sparrows. Only data points after the peak (age = 3.4) and only linear effects were included. Levels and corresponding sample sizes for categorical effects: natal environment: noisy = 21, quiet = 141; rearing environment: noisy = 36, quiet = 126; sex: female = 68, male = 94.

| **Fixed effects** | **Level** | **Estimate** | **Std. Error** | **z** | **p** |
| --- | --- | --- | --- | --- | --- |
| (Intercept) |  | 1.480 | 1.166 | 1.270 | 0.204 |
| Age |  | -0.255 | 0.302 | -0.844 | 0.398 |
| Natal environment | Quiet | -0.274 | 0.568 | -0.482 | 0.629 |
| Rearing environment | Quiet | -0.741 | 0.497 | -1.489 | 0.136 |
| Number of hatchlings |  | 0.073 | 0.207 | 0.352 | 0.725 |
| Number of fledglings |  | -0.056 | 0.212 | -0.262 | 0.793 |
| Sex | Male | 0.011 | 0.362 | 0.029 | 0.977 |
| Natal brood order |  | 0.145 | 0.389 | 0.373 | 0.709 |
| Foster brood order |  | -0.052 | 0.354 | -0.147 | 0.883 |
| **Random effects** | **162 observations** | **Variance** |  |  |  |
| Bird ID | 84 individuals | <0.001 |  |  |  |
| Year | 19 years | 0.471 |  |  |  |
| Natal brood ID | 71 natal broods | 0.018 |  |  |  |
| Foster brood ID | 71 foster broods | 0.018 |  |  |  |

**Table S3** Estimates from the full GLMM (before removing insignificant interactions) explaining the number of annual genetic recruits by age in adult Lundy house sparrows. Levels and corresponding sample sizes for categorical effects: natal environment: noisy = 53 (female) and 58 (male), quiet = 221 (female) and 298 (male); rearing environment: noisy = 69 (female) and 58 (male), quiet = 205 (female) and 298 (male); last reproduction: no = 159 (female) and 222 (male), yes = 115 (female) and 134 (male). Significant highest-order fixed effects are in bold. Δage: within-individual-centered age.

| **Fixed effects** | **Level** | **Estimate** | | **Std. Error** | **z** | **P** | **Estimate** | | **Std. Error** | **z** | **P** |
| --- | --- | --- | --- | --- | --- | --- | --- | --- | --- | --- | --- |
|  |  |  | **Female** | | | |  | **Male** | | | |
| (Intercept) |  | -3.634 | | 0.855 | -4.249 | <0.001 | -2.000 | | 0.851 | -2.350 | 0.019 |
| Number of hatchlings |  | 0.254 | | 0.119 | 2.136 | 0.033 | 0.016 | | 0.113 | 0.142 | 0.887 |
| Number of fledglings |  | -0.080 | | 0.122 | -0.655 | 0.512 | 0.058 | | 0.125 | 0.463 | 0.643 |
| Natal environment | Quiet | 0.244 | | 0.527 | 0.463 | 0.644 | -0.660 | | 0.616 | -1.071 | 0.284 |
| Rearing environment | Quiet | 0.188 | | 0.598 | 0.314 | 0.754 | 0.318 | | 0.580 | 0.548 | 0.583 |
| Δage |  | -0.468 | | 0.336 | -1.392 | 0.164 | -0.531 | | 0.392 | -1.356 | 0.175 |
| Δage^2^ |  | -0.358 | | 0.227 | -1.577 | 0.115 | -0.544 | | 0.293 | -1.858 | 0.063 |
| Natal brood order |  | 0.048 | | 0.178 | 0.268 | 0.789 | -0.043 | | 0.190 | -0.226 | 0.821 |
| Foster brood order |  | 0.113 | | 0.169 | 0.669 | 0.503 | -0.160 | | 0.174 | -0.925 | 0.355 |
| Last reproduction | Yes | 0.355 | | 0.227 | 1.567 | 0.117 | 0.331 | | 0.249 | 1.328 | 0.184 |
| Mean age |  | **0.756** | | **0.146** | **5.173** | **<0.001** | **0.787** | | **0.166** | **4.749** | **<0.001** |
| Number of fledglings × Δage |  | 0.074 | | 0.090 | 0.822 | 0.411 | -0.033 | | 0.097 | -0.343 | 0.731 |
| Natal environment × Δage | Quiet | -0.035 | | 0.309 | -0.114 | 0.909 | 0.092 | | 0.333 | 0.275 | 0.783 |
| Rearing environment × Δage | Quiet | 0.189 | | 0.207 | 0.916 | 0.360 | 0.709 | | 0.313 | 2.264 | 0.024 |
| Number of fledglings × Δage^2^ |  | 0.023 | | 0.073 | 0.321 | 0.748 | 0.012 | | 0.075 | 0.158 | 0.874 |
| Natal environment × Δage^2^ | Quiet | 0.219 | | 0.203 | 1.082 | 0.279 | 0.391 | | 0.297 | 1.316 | 0.188 |
| Rearing environment × Δage^2^ | Quiet | -0.165 | | 0.131 | 0.935 | 0.209 | -0.129 | | 0.224 | -0.578 | 0.563 |
| Natal environment × Rearing environment | Quiet × Quiet | 0.624 | | 0.667 |  | 0.350 | 0.358 | | 0.681 | 0.525 | 0.600 |
| **Random effects** |  | **274 observations** | |  |  | **Variance** | **356 observations** | |  |  | **Variance** |
| Bird ID |  | 133 individuals | |  |  | <0.001 | 165 individuals | |  |  | 0.186 |
| Year |  | 20 years | |  |  | 0.296 | 21 years | |  |  | 0.563 |
| Natal brood ID |  | 108 natal broods | |  |  | <0.001 | 148 natal broods | |  |  | <0.001 |
| Foster brood ID |  | 111 foster broods | |  |  | 0.115 | 148 foster broods | |  |  | <0.001 |

**Table S4** Estimates from the GLMM explaining the number of annual genetic recruits by age in female house sparrows, with only one bird randomly sampled from each natal brood. Levels and corresponding sample sizes for categorical effects: natal environment: noisy = 48, quiet = 184; rearing environment: noisy = 60, quiet = 172; last reproduction (LR): no = 137, yes = 95. Significant highest-order fixed effects are in bold. Δage: within-individual-centered age.

| **Fixed effects** | **Level** | **Estimate** | | **Std. Error** | **z** | **P** |
| --- | --- | --- | --- | --- | --- | --- |
|  |  |  | **Female** | | | |
| (Intercept) |  | -4.111 | | 0.836 | -4.916 | <0.001 |
| Number of hatchlings |  | **0.244** | | **0.118** | **2.067** | **0.039** |
| Number of fledglings |  | -0.024 | | 0.111 | -0.218 | 0.827 |
| Natal environment | Quiet | **0.900** | | **0.340** | **2.646** | **0.008** |
| Rearing environment | Quiet | 0.392 | | 0.257 | 1.526 | 0.127 |
| Δage |  | -0.167 | | 0.104 | -1.609 | 0.108 |
| Δage^2^ |  | **-0.117** | | **0.055** | **-2.145** | **0.032** |
| Natal brood order |  | 0.052 | | 0.176 | 0.294 | 0.769 |
| Foster brood order |  | 0.123 | | 0.170 | 0.724 | 0.469 |
| **Last reproduction** | **Yes** | **0.478** | | **0.237** | **2.017** | **0.044** |
| Mean age |  | **0.702** | | **0.147** | **4.759** | **<0.001** |
| **Random effects** |  | **232 observations** | |  |  | **Variance** |
| Bird ID |  | 108 individuals | |  |  | 0.008 |
| Year |  | 20 years | |  |  | 0.338 |
| Foster brood ID |  | 108 foster broods | |  |  | 0.008 |

**Table S5** Estimates from the GLMM explaining annual adult survival by age in Lundy house sparrows, with two extraordinarily long-lived individuals excluded (one male and one female). Levels and corresponding sample sizes for categorical effects: natal environment: noisy = 351, quiet = 1,316; rearing environment: noisy = 339, quiet = 1,328; sex: female = 787, male = 880. Significant highest-order fixed effects are in bold. Natal brood ID was removed from the random effects because it caused a convergence problem.

| **Fixed effects** | **Level** | **Estimate** | **Std. Error** | **z** | **P** |
| --- | --- | --- | --- | --- | --- |
| (Intercept) |  | -0.390 | 0.328 | -1.187 | 0.235 |
| Age |  | 0.412 | 0.208 | 1.984 | 0.047 |
| Age^2^ |  | -0.078 | 0.075 | -1.035 | 0.300 |
| Natal environment | Quiet | 0.352 | 0.196 | 1.793 | 0.073 |
| Rearing environment | Quiet | 0.132 | 0.157 | 0.842 | 0.400 |
| Number of hatchlings |  | 0.034 | 0.069 | 0.498 | 0.618 |
| Number of fledglings |  | -0.077 | 0.071 | -1.090 | 0.276 |
| **Sex** | **Male** | **0.267** | **0.118** | **2.259** | **0.024** |
| Natal brood order |  | -0.094 | 0.101 | -0.933 | 0.351 |
| Foster brood order |  | -0.077 | 0.100 | -0.763 | 0.446 |
| Age × Natal environment |  | 0.393 | 0.220 | 1.789 | 0.074 |
| **Age × Number of fledglings** |  | **0.155** | **0.059** | **2.620** | **0.009** |
| **Age^2^ × Natal environment** |  | **-0.236** | **0.088** | **-2.667** | **0.008** |
| **Random effects** | **1,667 observations** | **Variance** |  |  |  |
| **Bird ID** | **1,055 individuals** | **<0.001** |  |  |  |
| Year | 19 years | 0.539 |  |  |  |
| Foster brood ID | 448 foster broods | 0.140 |  |  |  |

**Table S6** Estimates from the GLMM explaining the number of annual genetic recruits by age in female and then male Lundy house sparrows, with two extraordinarily long-lived individuals excluded (one male and one female). Levels and corresponding sample sizes for categorical effects: natal environment: noisy = 53 (female) and 58 (male), quiet = 212 (female) and 289 (male); rearing environment: noisy = 60 (female) and 49 (male), quiet = 205 (female) and 298 (male); last reproduction (LR): no = 151 (female) and 214 (male), yes = 114 (female) and 133 (male). Significant highest-order fixed effects are in bold. Natal Brood ID was removed from the random effects in the female model because it caused a convergence problem. Δage: within-individual-centered age.

| **Fixed effects** | **Level** | **Estimate** | | **Std. Error** | **z** | **P** | **Estimate** | | **Std. Error** | **z** | **P** |
| --- | --- | --- | --- | --- | --- | --- | --- | --- | --- | --- | --- |
|  |  |  | **Female** | | | |  | **Male** | | | |
| (Intercept) |  | -3.967 | | 0.823 | -4.817 | <0.001 | -2.335 | | 0.709 | -3.295 | 0.001 |
| Number of hatchlings |  | 0.226 | | 0.117 | 1.933 | 0.053 | 0.015 | | 0.105 | 0.145 | 0.885 |
| Number of fledglings |  | -0.088 | | 0.110 | -0.802 | 0.423 | 0.003 | | 0.107 | 0.027 | 0.978 |
| Natal environment | Quiet | **0.895** | | **0.360** | **2.485** | **0.013** | -0.011 | | 0.267 | -0.043 | 0.966 |
| Rearing environment | Quiet | 0.327 | | 0.267 | 1.226 | 0.220 | 0.036 | | 0.296 | 0.120 | 0.904 |
| Δage |  | -0.131 | | 0.119 | -1.097 | 0.273 | 0.041 | | 0.125 | 0.328 | 0.743 |
| Δage^2^ |  | **-0.287** | | **0.087** | **-3.282** | **0.001** | **-0.400** | | **0.090** | **-4.421** | **<0.001** |
| Natal brood order |  | 0.008 | | 0.176 | 0.045 | 0.964 | 0.005 | | 0.179 | 0.028 | 0.978 |
| Foster brood order |  | 0.087 | | 0.169 | 0.512 | 0.608 | -0.160 | | 0.160 | -0.999 | 0.318 |
| Last reproduction | Yes | **0.478** | | **0.233** | **2.054** | **0.040** | **0.489** | | **0.248** | **1.972** | **0.049** |
| Mean age |  | **0.949** | | **0.175** | **5.427** | **<0.001** | **1.084** | | **0.175** | **6.201** | **<0.001** |
| **Random effects** |  | **274 observations** | |  |  | **Variance** | **347 observations** | |  |  | **Variance** |
| Bird ID |  | 133 individuals | |  |  | <0.001 | 164 individuals | |  |  | 0.094 |
| Year |  | 18 years | |  |  | 0.351 | 16 years | |  |  | 0.493 |
| Natal brood ID |  |  | |  |  |  | 147 natal broods | |  |  | <0.001 |
| Foster brood ID |  | 111 foster broods | |  |  | 0.111 | 147 foster broods | |  |  | <0.001 |

**Table S7** Number of individuals by their cross-fostering status and sex. Cross-fostering status is represented by their natal environment (quiet/noisy) and rearing environment (quiet/noisy) connected by a “→”.

| Sex  Cross-fostering status | Female | Male |
| --- | --- | --- |
| Quiet → quiet | 315 | 335 |
| Quiet → noisy | 81 | 86 |
| Noisy → quiet | 92 | 83 |
| Noisy → noisy | 35 | 30 |

**Table S8** Summary of non-significant interaction terms removed during model simplification. Estimates, standard errors, z statistics, and p-values are reported for each interaction term dropped from the initial full model of annual survival. Terms were sequentially removed in order of increasing p-value, retaining only significant interactions in the final model (see Methods).

*(uploaded as a separate .xlsx file due to the size of the table)*

**Table S9** Summary of non-significant interaction terms removed during model simplification. Estimates, standard errors, z statistics, and p-values are reported for each interaction term dropped from the initial full model of female annual reproductive output. Terms were sequentially removed in order of increasing p-value, retaining only significant interactions in the final model (see Methods).

*(uploaded as a separate .xlsx file due to the size of the table)*

**Table S10** Summary of non-significant interaction terms removed during model simplification. Estimates, standard errors, z statistics, and p-values are reported for each interaction term dropped from the initial full model of male annual reproductive output. Terms were sequentially removed in order of increasing p-value, retaining only significant interactions in the final model (see Methods).

*(uploaded as a separate .xlsx file due to the size of the table)*


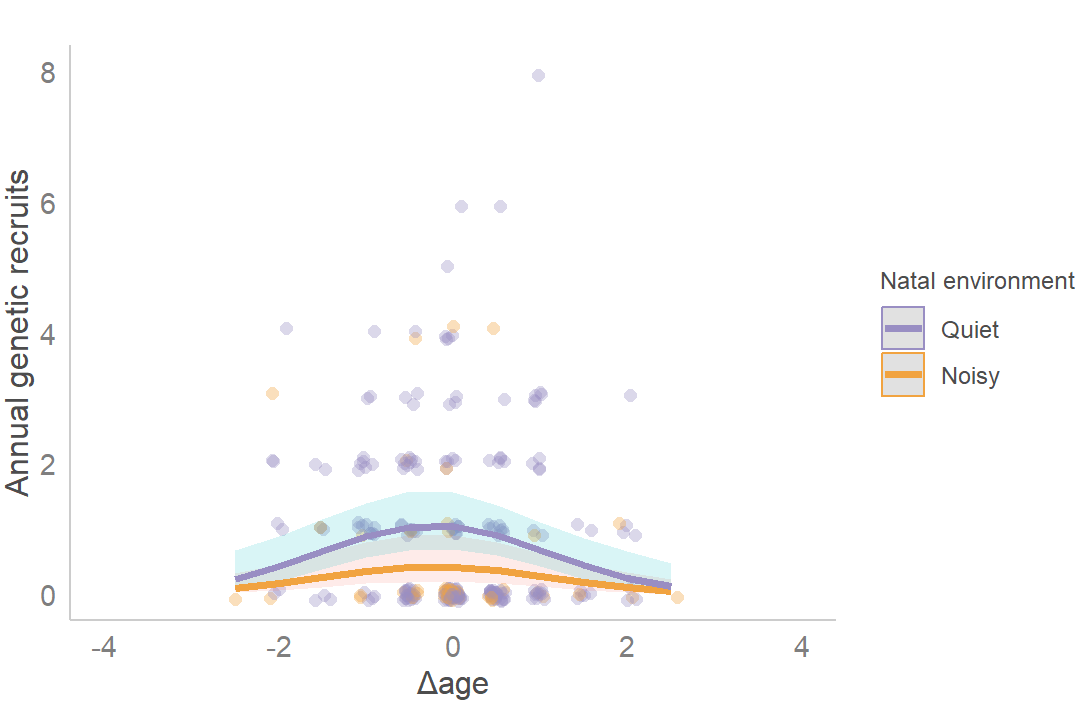


**Figure S1** The number of annual genetic recruits in relationship to within-individual-centered age of adult female Lundy house sparrows, excluding the one exceptionally long-lived female. The mean ages of birds hatched in a noisy (orange) and quiet (purple) environment were: 1.8 y and 1.9 y. Each dot represents an observation; lines are predicted number of annual genetic recruits; shades represent 95% confidence intervals.
